# Respiratory Oscillometry Findings in Chronic Epipharyngitis

**DOI:** 10.3390/reports9030276

**Published:** 2026-08-19

**Authors:** Manabu Mogitate

**Affiliations:** Mogitate ENT Clinic, Kawasaki 213-011, Kanagawa, Japan; mogitateentjp@m08.itscom.net

**Keywords:** chronic nasopharyngeal inflammation, chronic epipharyngitis, Epipharyngeal Abrasive Therapy, respiratory oscillometry, forced oscillation technique, MostGraph, small airway dysfunction, respiratory reactance, X5, Fres, ALX

## Abstract

Background: Chronic nasopharyngeal inflammation (chronic epipharyngitis) is associated with various upper airway and systemic symptoms. However, the relationship between endoscopic inflammatory findings and functional respiratory abnormalities remains unclear. This study aimed to evaluate respiratory oscillometry parameters measured by the MostGraph system in patients with chronic epipharyngitis and to investigate their changes following Epipharyngeal Abrasive Therapy (EAT). Methods: In this retrospective observational study, patients diagnosed with chronic epipharyngitis who underwent MostGraph examination were analyzed. Respiratory resistance and reactance parameters, including R5−R20, X5, inspiratory X5 (Xin5), resonant frequency (Fres), and low-frequency reactance area (ALX), were assessed. Endoscopic findings were scored using a standardized grading system. Comparisons were performed between patients and healthy controls, and pre- and post-EAT measurements were evaluated. Correlations between endoscopic severity and oscillometric parameters were analyzed using Spearman’s rank correlation coefficient. Results: Patients with chronic epipharyngitis demonstrated significant abnormalities in oscillometric reactance parameters compared with healthy controls. Following EAT, a significant change was observed in R5−R20, whereas other oscillometric parameters did not show significant changes. In addition, endoscopic severity scores showed no significant correlation with oscillometric measurements. These findings suggest that respiratory oscillometry may detect physiological abnormalities that are not directly reflected by endoscopic inflammatory severity. Conclusions: Patients with chronic epipharyngitis exhibited abnormalities in respiratory oscillometry parameters despite the absence of significant associations with endoscopic severity scores. The observed dissociation between endoscopic findings and oscillometric measurements suggests that morphological inflammation and physiological dysfunction may represent distinct dimensions of the disease. Respiratory oscillometry may therefore provide complementary information regarding the pathophysiology of chronic epipharyngitis beyond conventional endoscopic assessment.

## 1. Introduction

Chronic nasopharyngeal inflammation (chronic epipharyngitis) is a chronic inflammatory disorder of the nasopharyngeal mucosa associated with a variety of local symptoms, including throat discomfort, postnasal drip, excessive sputum production, and chronic cough. In the 1960s, Horiguchi and colleagues proposed an association between chronic nasopharyngitis and systemic diseases and emphasized its potential clinical significance. Despite its long history in Japan, chronic epipharyngitis remains insufficiently recognized internationally, and both its pathophysiology and objective assessment methods remain incompletely understood [1]. Clinically, the diagnosis of chronic epipharyngitis in Japan is generally based on characteristic endoscopic findings and the presence of contact bleeding induced by epipharyngeal abrasion. Endoscopic evaluation typically assesses mucosal redness, swelling, mucus or crust adhesion, and bleeding, which are commonly used indicators of disease severity [2,3]. Recent studies have suggested that chronic epipharyngitis may not be restricted to the upper airway but may also be associated with systemic disorders, including IgA nephropathy, myalgic encephalomyelitis/chronic fatigue syndrome (ME/CFS), and post-COVID-19 condition (long COVID). These observations have increased interest in the potential interactions among chronic epipharyngitis, immune responses, autonomic nervous system regulation, and systemic inflammatory pathways [4,5,6,7,8,9,10]. The etiology of chronic epipharyngitis remains incompletely understood and is likely multifactorial. Various local and systemic factors have been proposed to contribute to its development; however, no single causative mechanism has been established. Persistent exposure to these factors may contribute to chronic mucosal inflammation and epithelial injury. In addition, recent studies have demonstrated persistent local immune activation characterized by altered mucosal immune responses, including Th17/Treg imbalance and increased expression of proinflammatory cytokines. These pathophysiological mechanisms may contribute to both local inflammatory findings and systemic manifestations associated with the disease [7,8,11].

Epipharyngeal Abrasive Therapy (EAT) is widely performed in Japan as a treatment for chronic epipharyngitis. In a specialty outpatient clinic, EAT was associated with symptomatic improvement in the majority of patients, with approximately 86% showing improvement or better outcomes. Endoscopic findings also improved during the treatment course in many patients [2]. Furthermore, Ohno and colleagues proposed an endoscopic grading system based on mucosal redness, swelling, mucus adhesion, and bleeding during abrasion, providing a structured and reproducible method for assessing the severity of chronic epipharyngitis [3].

Recently, fractional exhaled nitric oxide (FeNO) has been investigated as a potential objective biomarker for chronic epipharyngitis. We previously demonstrated that FeNO levels decreased following EAT and were associated with treatment response. Furthermore, FeNO levels were positively correlated with endoscopic severity scores, suggesting that FeNO reflects inflammatory activity within the epipharyngeal mucosa. These findings support the utility of FeNO as an objective marker of disease severity and treatment response [12,13]. Recent histopathological studies have further demonstrated that chronic epipharyngitis is characterized by persistent local immune activation and increased expression of proinflammatory cytokines, including interleukin-6 (IL-6) and tumor necrosis factor-α (TNF-α), both of which are suppressed following EAT [9,10].

In contrast, little is known about functional respiratory abnormalities in patients with chronic epipharyngitis. The Forced Oscillation Technique (FOT) is a noninvasive method for evaluating respiratory mechanics during tidal breathing and enables assessment of respiratory reactance parameters, including reactance at 5 Hz (X5), inspiratory X5 (Xin5), resonant frequency (Fres), and low-frequency reactance area (ALX). International technical standards have established these parameters as important indicators of peripheral airway dysfunction and respiratory mechanical abnormalities in various respiratory diseases. In contrast to conventional spirometry, which requires forced respiratory maneuvers, FOT can be performed during quiet tidal breathing and is considered particularly useful for detecting subtle physiological abnormalities that may not be evident on routine pulmonary function testing. Recent studies have demonstrated that oscillometric reactance indices are sensitive markers of small-airway dysfunction, ventilation heterogeneity, and altered respiratory mechanics in asthma and other airway disorders [14,15,16,17].

However, respiratory reactance abnormalities have not previously been investigated in patients with chronic epipharyngitis. Because some patients with chronic epipharyngitis complain of respiratory discomfort despite unremarkable findings on routine examinations, respiratory oscillometry may provide complementary information regarding functional abnormalities that are not captured by conventional assessments.

Therefore, the present study aimed to evaluate respiratory reactance parameters using MostGraph^®^ in patients with chronic epipharyngitis, to investigate changes following EAT, and to determine the relationship between respiratory reactance and endoscopic inflammatory severity. Previous studies have suggested that objective assessments such as FeNO primarily reflect inflammatory activity within the epipharyngeal mucosa, whereas little is known about physiological abnormalities related to respiratory function. We hypothesized that respiratory reactance may represent a functional dimension of chronic epipharyngitis distinct from the morphological inflammatory findings observed by endoscopy and the inflammatory activity reflected by FeNO.

## 2. Methods

### 2.1. Study Design and Participants

This retrospective observational study was conducted at Mogitate ENT Clinic, Kawasaki, Japan. Patients diagnosed with chronic epipharyngitis who underwent respiratory oscillometry using the MostGraph^®^ system between January 2022 and February 2022 were enrolled consecutively. Inclusion criteria were a diagnosis of chronic epipharyngitis, availability of baseline respiratory oscillometry data, and completion of 12 sessions of EAT with follow-up oscillometric assessment. Exclusion criteria included incomplete clinical data, absence of follow-up measurements, and inability to complete the planned treatment protocol.

The diagnosis of chronic epipharyngitis was established based on endoscopic findings and the presence of bleeding during epipharyngeal abrasion. Patients who completed 12 sessions of EAT and underwent respiratory oscillometry both before treatment initiation and after 12 treatment sessions were included in the analysis.

A healthy control group consisting of volunteers without a history of chronic respiratory disease, bronchial asthma, chronic rhinosinusitis, or chronic epipharyngitis was also evaluated for comparison. Information regarding smoking status, allergic diseases, respiratory comorbidities, and medication use was collected whenever available.

**Figure 1:**
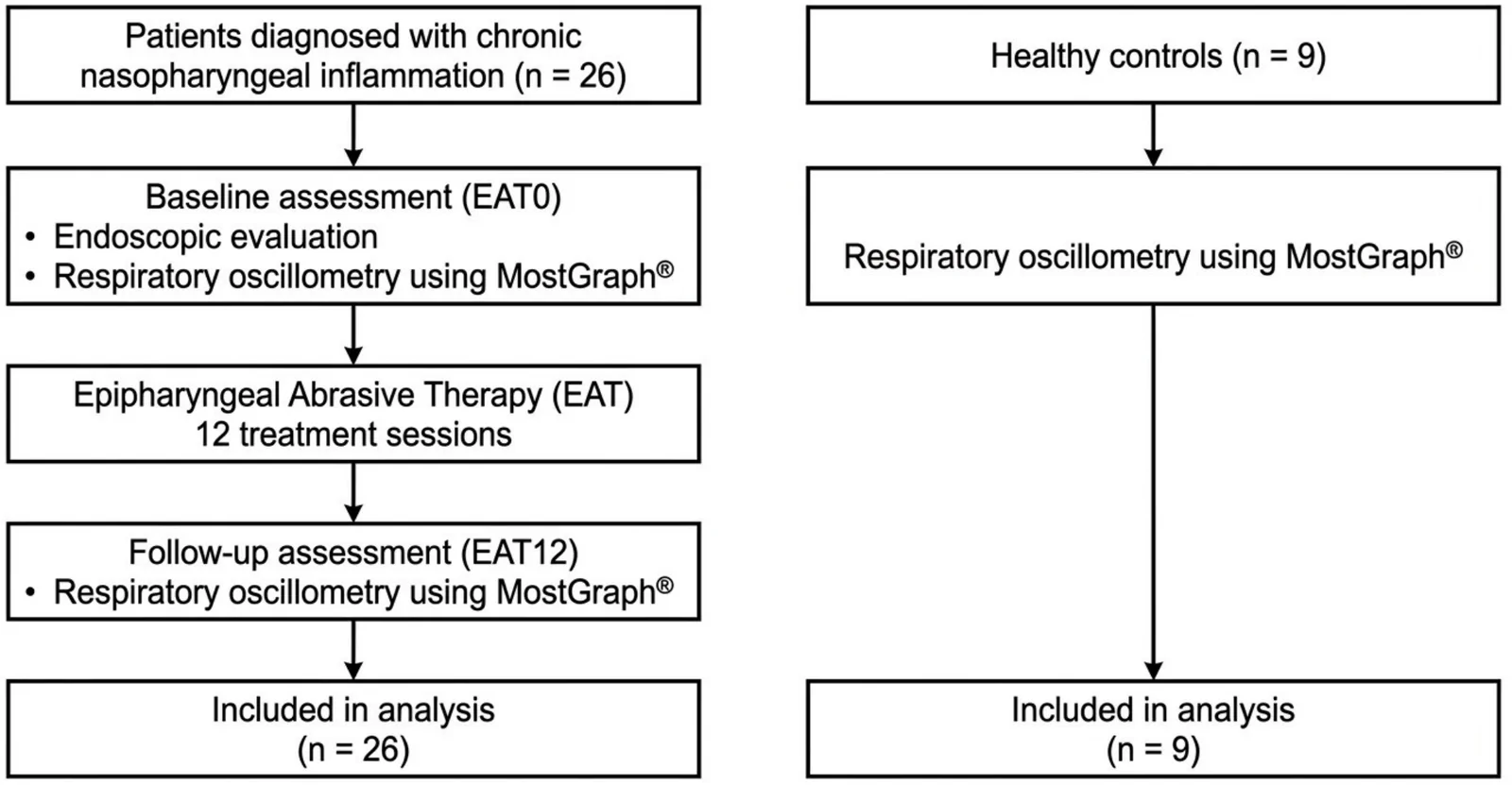
Study Flow Diagram. Flow diagram of participant selection and study procedures. Twenty-six patients with chronic epipharyngitis who underwent respiratory oscillometry using the MostGraph^®^ system and completed 12 sessions of EAT were included in the analysis. Nine healthy volunteers were enrolled as controls for baseline comparison.

The study was approved by the Ethics Committee of Ota General Hospital (Approval No. 22027; approved on 20 October 2022). Patient consent was waived by the Ethics Committee because of the retrospective nature of the study. An opt-out procedure was used to provide participants with the opportunity to refuse participation. The study was conducted in accordance with the Declaration of Helsinki. A flow diagram of patient selection and study procedures is shown in Figure 1.

### 2.2. Epipharyngeal Abrasive Therapy

EAT was performed according to the standard method used in Japan. [2,3] Following topical anesthesia of the nasal cavity, the nasopharyngeal mucosa was mechanically abraded using a cotton swab soaked in 1% zinc chloride solution.

Treatment was performed once weekly. Respiratory oscillometry was performed at two predefined time points: immediately before the first EAT session at the initial visit (EAT0) and after completion of 12 EAT sessions (EAT12).

### 2.3. Endoscopic Evaluation

**Figure 2:**
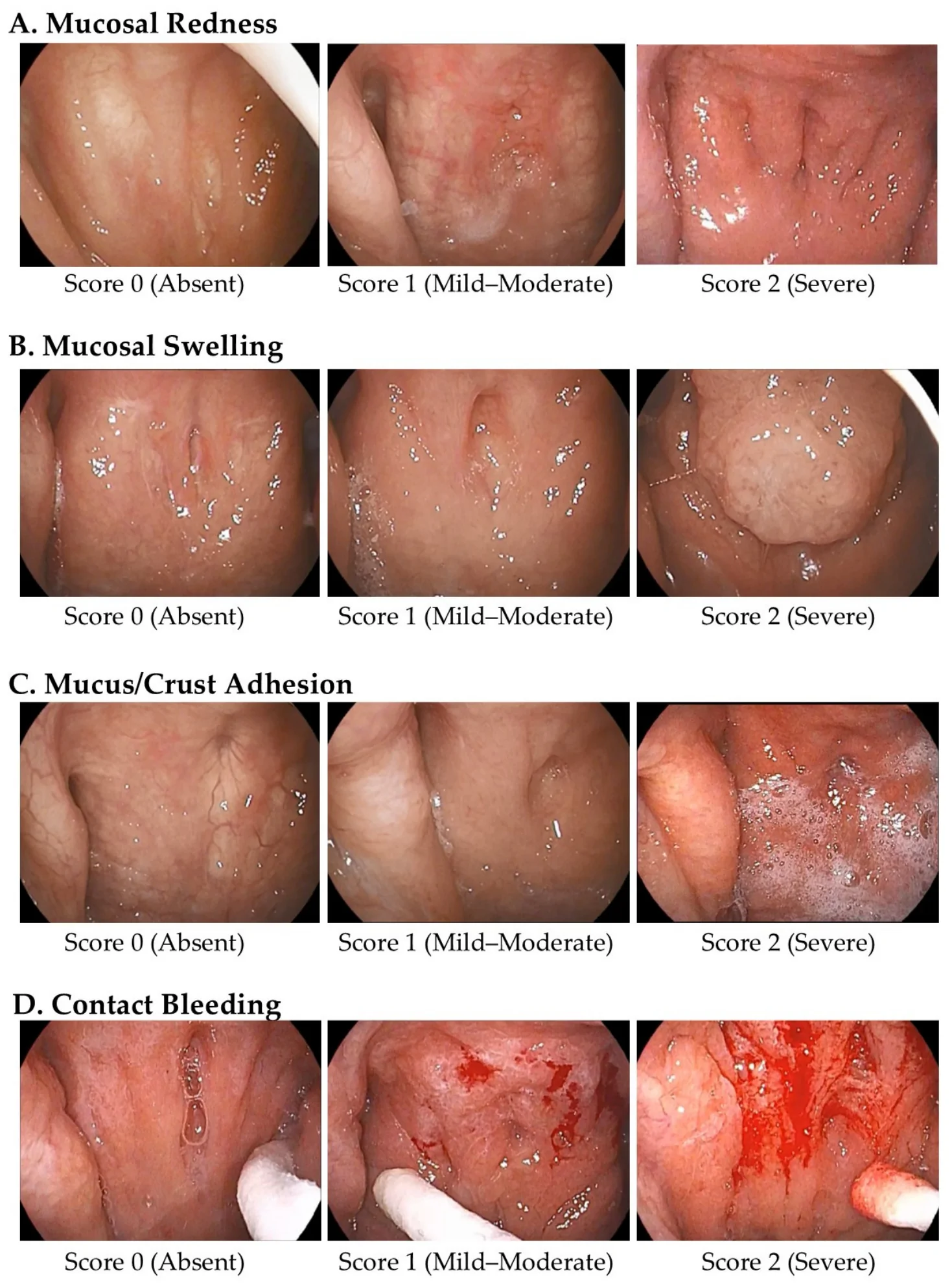
Representative Endoscopic Findings Used for Severity Grading of Chronic Epipharyngitis. Representative endoscopic images demonstrating the grading criteria used for assessment of chronic epipharyngitis. Mucosal redness, mucosal swelling, mucus/crust adhesion, and contact bleeding were each graded as 0 (absent), 1 (mild-to-moderate), or 2 (severe). The total endoscopic severity score was calculated as the sum of these four components and ranged from 0 to 8 points.

Endoscopic examinations were performed using a flexible transnasal endoscope (EPK-i7000 video processor and VNL-1190STK video nasoscope; HOYA Corporation, Tokyo, Japan). Endoscopic severity at baseline (EAT0) was evaluated according to the assessment criteria proposed by the Epipharyngeal Abrasive Therapy Review Committee. The degree of mucosal redness, mucosal swelling, mucus or crust adhesion, and bleeding induced by abrasion were each graded on a three-point scale (0 = absent, 1 = mild to moderate, and 2 = severe). The total endoscopic score ranged from 0 to 8 points, with higher scores indicating more severe inflammation. Representative endoscopic images illustrating each grading category are provided in Figure 2. Endoscopic assessments were performed by the treating physician at the time of examination and were not blinded to the clinical information or treatment status.

### 2.4. Respiratory Oscillometry

Respiratory oscillometry was performed using MostGraph^®^ (CHEST M.I., Inc., Tokyo, Japan), which is based on the Forced Oscillation Technique (FOT). Measurements were obtained during quiet tidal breathing with participants in the seated position while wearing a nose clip and supporting their cheeks with both hands in accordance with international technical standards.

Respiratory resistance parameters included resistance at 5 Hz (R5), resistance at 20 Hz (R20), and the difference between R5 and R20 (R5−R20), reflecting frequency-dependent resistance and peripheral airway dysfunction. Reactance parameters included reactance at 5 Hz (X5), inspiratory reactance at 5 Hz (Xin5), resonant frequency (Fres), and low-frequency reactance area (ALX). These parameters were selected because they are commonly used indices of respiratory mechanical function and peripheral airway abnormalities.

### 2.5. Outcome Measures

The primary outcome was the difference in respiratory oscillometry parameters between patients with chronic epipharyngitis and healthy controls.

Secondary outcomes included:

- Changes in respiratory resistance and reactance parameters between EAT0 and EAT12.
- Associations between baseline endoscopic severity scores and respiratory oscillometry parameters.
- Associations between baseline endoscopic severity scores and changes in respiratory oscillometry parameters following EAT.

### 2.6. Statistical Analysis

Continuous variables are presented as medians and interquartile ranges (IQRs). Comparisons between patients and healthy controls were performed using the Mann–Whitney U test. Changes between EAT0 and EAT12 were analyzed using the Wilcoxon signed-rank test. Associations between endoscopic severity scores and respiratory oscillometry parameters were evaluated using Spearman’s rank correlation coefficient.

All statistical analyses were performed using SPSS Statistics version 22.0 (IBM Corp., Armonk, NY, USA). Because this was an exploratory study, no formal correction for multiple comparisons was applied. Therefore, the findings should be interpreted with appropriate caution. A two-sided *p* value of less than 0.05 was considered statistically significant.

## 3. Results

### 3.1. Participant Characteristics

A total of 26 patients with chronic epipharyngitis and 9 healthy controls were included in the study. The median age was 40 years in both groups. The chronic epipharyngitis group consisted of 6 males and 20 females, whereas the healthy control group consisted of 2 males and 7 females. Participant characteristics, including available clinical background information, are summarized in Table 1.

### 3.2. Distribution of Baseline Endoscopic Severity Scores

The distribution of patients according to baseline endoscopic severity scores is summarized in Table 2. The most common endoscopic severity score was 5 (38.5%), followed by scores of 3 (30.8%) and 6 (19.2%).

### 3.3. Baseline Respiratory Oscillometry Parameters

**Figure 3:**
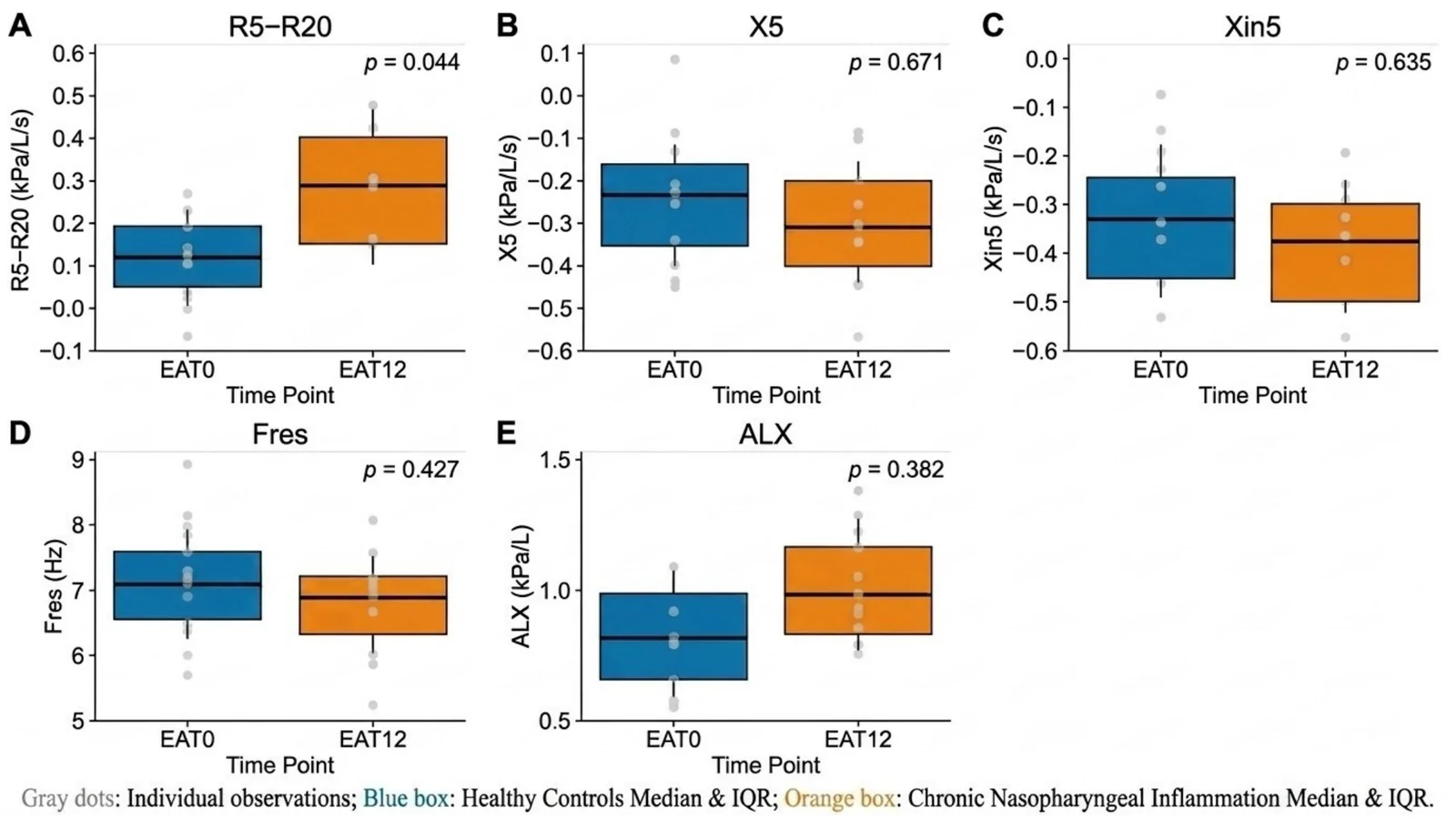
Baseline Respiratory Oscillometry Parameters in Healthy Controls and Patients with Chronic Epipharyngitis. (**A**) R5–R20 values at baseline. No significant difference was observed between groups. (**B**) X5 values at baseline. Patients showed significantly altered X5 compared with healthy controls. (**C**) Inspiratory Xn5 (Xin5) values at baseline. Patients demonstrated significantly altered Xin5. (**D**) Fres values at baseline. Fres was significantly higher in patients than in controls. (**E**) ALX values at baseline. ALX was significantly increased in patients. Box plots represent the median and interquartile range with individual observations overlaid. p values were calculated using the Mann–Whitney U test.

Baseline respiratory oscillometry parameters in healthy controls and patients with chronic epipharyngitis are shown in Table 3 and Figure 3.

Compared with healthy controls, patients with chronic epipharyngitis demonstrated significantly altered respiratory reactance parameters. X5 was significantly lower in patients than in controls (*p* = 0.034), and inspiratory X5 (Xin5) was also significantly lower in patients (*p* = 0.013). In addition, Fres and ALX were significantly higher in patients with chronic epipharyngitis than in healthy controls (*p* = 0.030 and *p* = 0.036, respectively). In contrast, no significant differences were observed in resistance parameters, including R5, R20, and R5−R20 (all *p* > 0.05).

### 3.4. Changes in Respiratory Oscillometry Parameters Following Epipharyngeal Abrasive Therapy

**Figure 4:**
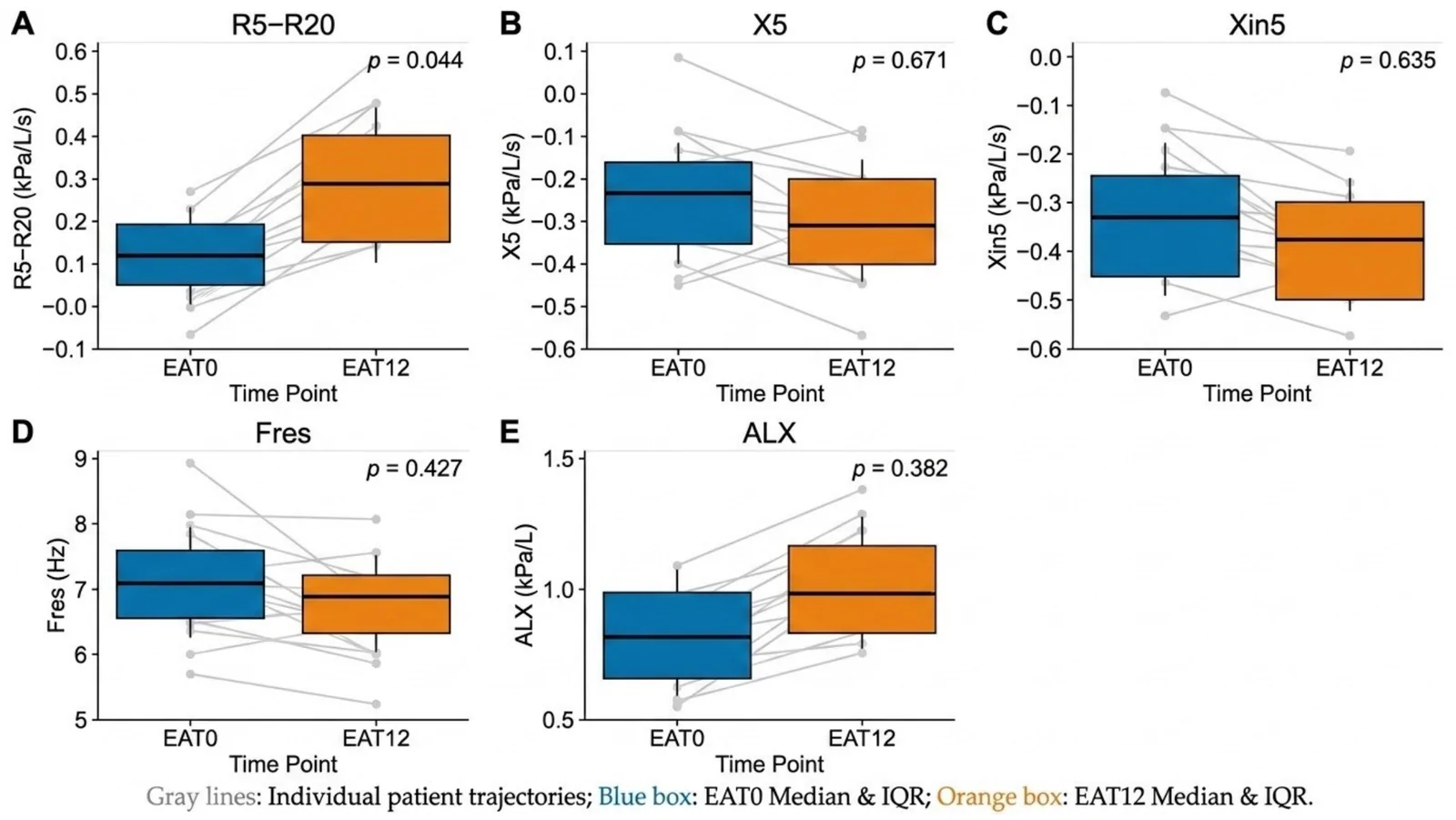
Changes in Respiratory Oscillometry Parameters Following Epipharyngeal Abrasive Therapy (EAT). (**A**) R5–R20 values between baseline (EAT0) and after 12 sessions of EAT (EAT12). (**B**) X5 values between EAT0 and EAT12. (**C**) Inspiratory Xins values between EAT0 and EAT12. (**D**) Fres values between EAT0 and EAT12. (**E**) ALX values between EAT0 and EAT12. Individual paired observations are connected by lines.

Changes in respiratory oscillometry parameters following EAT are summarized in Table 4 and Figure 4.

Among the evaluated parameters, R5−R20 showed a significant change following EAT (median: 0.12 at EAT0 vs. 0.29 at EAT12, *p* = 0.044). In contrast, no significant changes were observed in X5, Xin5, Fres, or ALX following treatment (all *p* > 0.05). Similarly, R5 and R20 did not significantly change between EAT0 and EAT12.

### 3.5. Correlations Between Endoscopic Severity Scores and Respiratory Oscillometry Parameters

**Figure 5:**
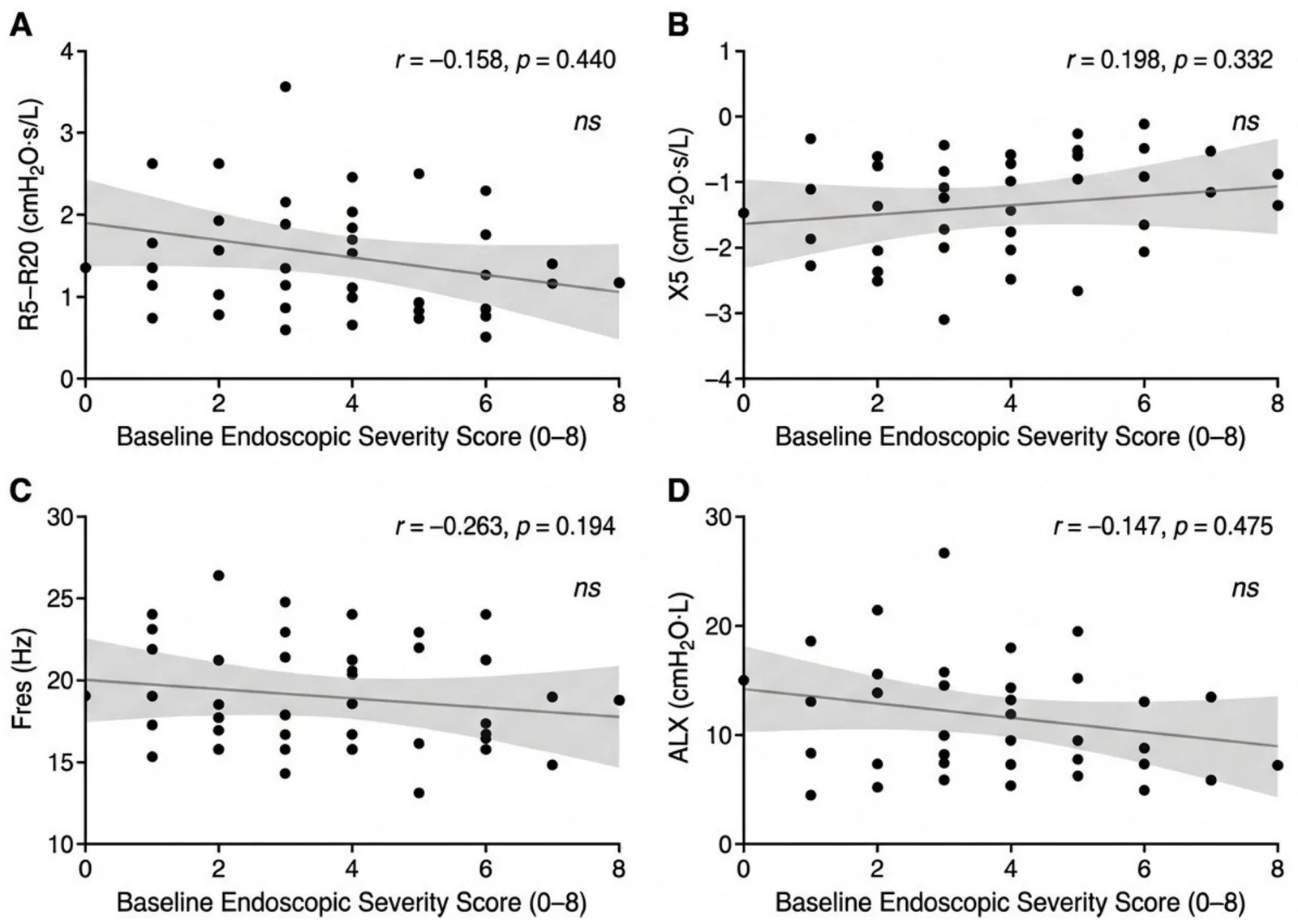
Relationship between Baseline Endoscopic Severity Scores and Respiratory Oscillometry Parameters. (**A**) Relationship between baseline endoscopic severity scores and R5–R20. Individual data points are shown as dots, with linear regression lines and 95% confidence intervals. (**B**) Relationship between baseline endoscopic severity scores and X5. Dots represent individual observations; regression lines with 95% confidence intervals are displayed. (**C**) Relationship between baseline endoscopic severity scores and Fres. Individual observations are shown as dots, with regression lines and 95% confidence intervals. (**D**) Relationship between baseline endoscopic severity scores and AX. Dots represent individual observations; regression lines with 95% confidence intervals are displayed. Spearman correlation coefficients (r) and *p* values are shown in each panel.

The relationships between baseline endoscopic severity scores and respiratory oscillometry parameters are shown in Table 5 and Figure 5.

Spearman correlation analysis revealed no significant correlations between baseline endoscopic severity scores and any respiratory oscillometry parameter. Specifically, no significant associations were observed for R5−R20 (r = −0.158, *p* = 0.440), X5 (r = 0.198, *p* = 0.332), Fres (r = −0.263, *p* = 0.194), or ALX (r = −0.147, *p* = 0.475). Likewise, no significant correlations were found for R5, R20, or Xin5 (all *p* > 0.05).

## 4. Discussion

To our knowledge, this is the first study to evaluate respiratory oscillometry findings in patients with chronic epipharyngitis and to assess changes following EAT. The present study demonstrated three principal findings. First, patients with chronic epipharyngitis exhibited significant abnormalities in oscillometric reactance parameters, including X5, inspiratory X5 (Xin5), Fres, and ALX, compared with healthy controls. Second, R5−R20 changed significantly following EAT. Third, no significant correlations were observed between endoscopic severity scores and oscillometric parameters.

Respiratory oscillometry has emerged as a sensitive and effort-independent method for assessing respiratory mechanics during tidal breathing. Recent reviews have emphasized that oscillometric reactance indices, including X5 and AX/ALX, reflect peripheral airway function and may detect abnormalities that are not apparent on conventional spirometry. Oscillometry is particularly sensitive to dysfunction of the small airways, often referred to as the “silent zone” of the lung. Unlike conventional spirometry, which requires forced respiratory maneuvers, oscillometry can be performed during quiet tidal breathing and therefore provides a practical and reproducible assessment of respiratory mechanics in routine clinical practice. Recent studies have shown that abnormalities in oscillometric reactance parameters are associated with small-airway dysfunction, ventilation heterogeneity, symptom burden, and poor disease control in patients with asthma and other chronic airway diseases [14,15,16,17].

In the present study, patients with chronic epipharyngitis demonstrated significantly lower X5 and Xin5 values and significantly higher Fres and ALX values than healthy controls. These findings suggest the presence of altered respiratory mechanics despite the absence of clinically recognized lower-airway disease in most patients. Similar oscillometric abnormalities have been reported in asthma and chronic airway diseases, where impaired reactance is considered a marker of peripheral airway dysfunction [16,17]. Recent studies have further demonstrated that oscillometric abnormalities in X5, Fres, and AX/ALX are associated with small-airway dysfunction, poor asthma control, increased symptom burden, and a greater risk of future exacerbations, highlighting the clinical relevance of these parameters as sensitive indicators of distal airway abnormalities [16,18,19]. Accordingly, the oscillometric pattern observed in the present study may reflect subtle physiological impairment that is not evident on routine clinical assessment or conventional spirometry. Notably, the abnormalities observed in the present study were predominantly related to respiratory reactance rather than respiratory resistance. Reactance parameters are considered to reflect the elastic and inertial properties of the respiratory system and are particularly sensitive to abnormalities affecting the peripheral airways and lung tissue mechanics [14,15,19]. Therefore, the present findings may indicate subtle physiological dysfunction that is not readily detected by routine clinical examinations.

The observation that reactance abnormalities were present despite the absence of overt lower-airway disease raises the possibility that chronic epipharyngitis may be associated with functional respiratory alterations extending beyond the upper airway. The present findings therefore suggest that chronic inflammation localized to the epipharynx may be accompanied by physiological changes extending beyond its anatomical location.

The mechanism underlying these abnormalities remains speculative. One possible explanation is the “united airway” concept, which proposes that the upper and lower airways share common inflammatory and immunological pathways [20,21]. Chronic epipharyngitis is characterized by persistent mucosal inflammation and has been associated with various systemic inflammatory conditions, including IgA nephropathy, myalgic encephalomyelitis/chronic fatigue syndrome, and post-COVID-19 condition [4,5,6,7,8,9,10]. Recent histopathological and transcriptomic studies have demonstrated persistent immune activation within the epipharyngeal mucosa, including increased expression of proinflammatory cytokines such as IL-6 and activation of inflammatory signaling pathways [9,10]. The concept of a continuous airway inflammatory axis linking the upper and lower airway has been well established in allergic rhinitis and asthma [18,20,21]. Although chronic epipharyngitis differs from these conditions, inflammation localized to the epipharynx may influence respiratory mechanics through shared inflammatory, neural, and mucosal immune pathways. Consequently, the oscillometric abnormalities observed in the present study may represent a functional manifestation of airway interactions extending beyond the anatomical site of inflammation, although this hypothesis remains unproven and should be interpreted with caution.

EAT was associated with a statistically significant change in R5−R20, whereas no significant changes were observed in the reactance parameters that differed between patients and healthy controls. However, because multiple oscillometric parameters were analyzed without formal correction for multiple comparisons, this finding should be interpreted cautiously and regarded as exploratory until confirmed in larger studies. R5−R20 is generally regarded as an index of frequency-dependent respiratory resistance and is widely used as a surrogate marker of peripheral airway dysfunction and ventilation heterogeneity. Previous studies in patients with asthma have demonstrated that elevated R5−R20 is associated with small-airway dysfunction, poor disease control, and an increased symptom burden. Therefore, alterations in R5−R20 are commonly interpreted as reflecting changes in small-airway physiology rather than central airway function [15,16,17,18,19,22].

Interestingly, the observed increase in R5−R20 following EAT was not accompanied by significant changes in other oscillometric parameters. One possible explanation is that EAT may influence autonomic nervous system activity rather than directly improving lower-airway mechanics. Clinically, transient rhinorrhea and sneezing are occasionally observed after EAT and are often interpreted as manifestations of parasympathetic activation. Because parasympathetic neural pathways regulate airway smooth-muscle tone, autonomic modulation may have contributed to the observed change in R5−R20. However, this interpretation remains speculative and requires further investigation [7,23,24].

However, the oscillometric abnormalities identified at baseline in patients with chronic epipharyngitis were primarily observed in reactance parameters, including X5, Xin5, Fres, and ALX, whereas these parameters did not significantly change after treatment [14,15,19]. Accordingly, the observed change in R5−R20 should not be interpreted as direct evidence of improvement in lower-airway function. Rather, it may indicate that EAT influences physiological pathways distinct from those responsible for the reactance abnormalities observed in chronic epipharyngitis [15,19,22]. Because autonomic function was not directly assessed in the present study, this interpretation remains speculative and requires confirmation in future studies incorporating simultaneous evaluation of autonomic activity and respiratory oscillometry [7,23,24].

Another important finding was the absence of significant correlations between endoscopic severity scores and respiratory oscillometry parameters. Endoscopic findings are widely used for the diagnosis and assessment of chronic epipharyngitis, and previous studies have demonstrated their clinical utility for evaluating disease severity and treatment response following EAT [2,3].

Furthermore, recent studies have demonstrated that characteristic endoscopic findings of chronic epipharyngitis can be assessed with reasonable reproducibility and may serve as suitable targets for objective image-based evaluation using artificial intelligence [25,26].

In contrast, we previously reported that FeNO levels correlate with endoscopic severity scores and decrease following EAT, suggesting that FeNO reflects inflammatory activity within the epipharyngeal mucosa [12,13].

However, no significant correlations were observed between endoscopic severity scores and respiratory oscillometry parameters in the present study.

These findings suggest that respiratory oscillometry may capture physiological characteristics that are distinct from the inflammatory changes visualized by endoscopy. Accordingly, endoscopic assessment and respiratory oscillometry may provide complementary rather than overlapping information regarding chronic epipharyngitis.

These findings may have important implications for future research. The present results suggest that endoscopic findings, inflammatory biomarkers such as FeNO, and respiratory oscillometry may capture different dimensions of chronic epipharyngitis, including morphological inflammation, inflammatory activity, and physiological dysfunction [12,13]. Integrating these complementary assessments may provide a more comprehensive understanding of disease pathophysiology than any single modality alone.

Several limitations should be acknowledged. First, this was a single-center, exploratory pilot study with a relatively small sample size, and no formal sample size calculation was performed before study initiation. Therefore, the statistical power of the study may have been limited, increasing the risk of type II error and limiting the generalizability of the findings. Second, spirometry was not routinely performed as part of the clinical assessment protocol during the study period, and therefore corresponding data were not available for analysis. As a result, direct comparisons between oscillometric findings and conventional pulmonary function parameters could not be performed. This limitation restricts the interpretation of the physiological significance of the observed oscillometric abnormalities. Third, the mechanisms linking chronic epipharyngitis and oscillometric abnormalities remain uncertain. Furthermore, autonomic nervous system activity was not directly assessed in the present study, and therefore the proposed autonomic mechanism underlying the observed oscillometric changes remains speculative. Fourth, multiple oscillometric parameters were evaluated, and therefore the statistically significant finding for R5−R20 should be considered exploratory and interpreted with caution. In addition, information regarding smoking status, body mass index, allergic diseases, chronic sinonasal conditions, comorbidities, and medication use was not available for all participants. Therefore, the possibility of residual confounding cannot be excluded. Finally, the observational design precludes conclusions regarding causality.

**Figure 6:**
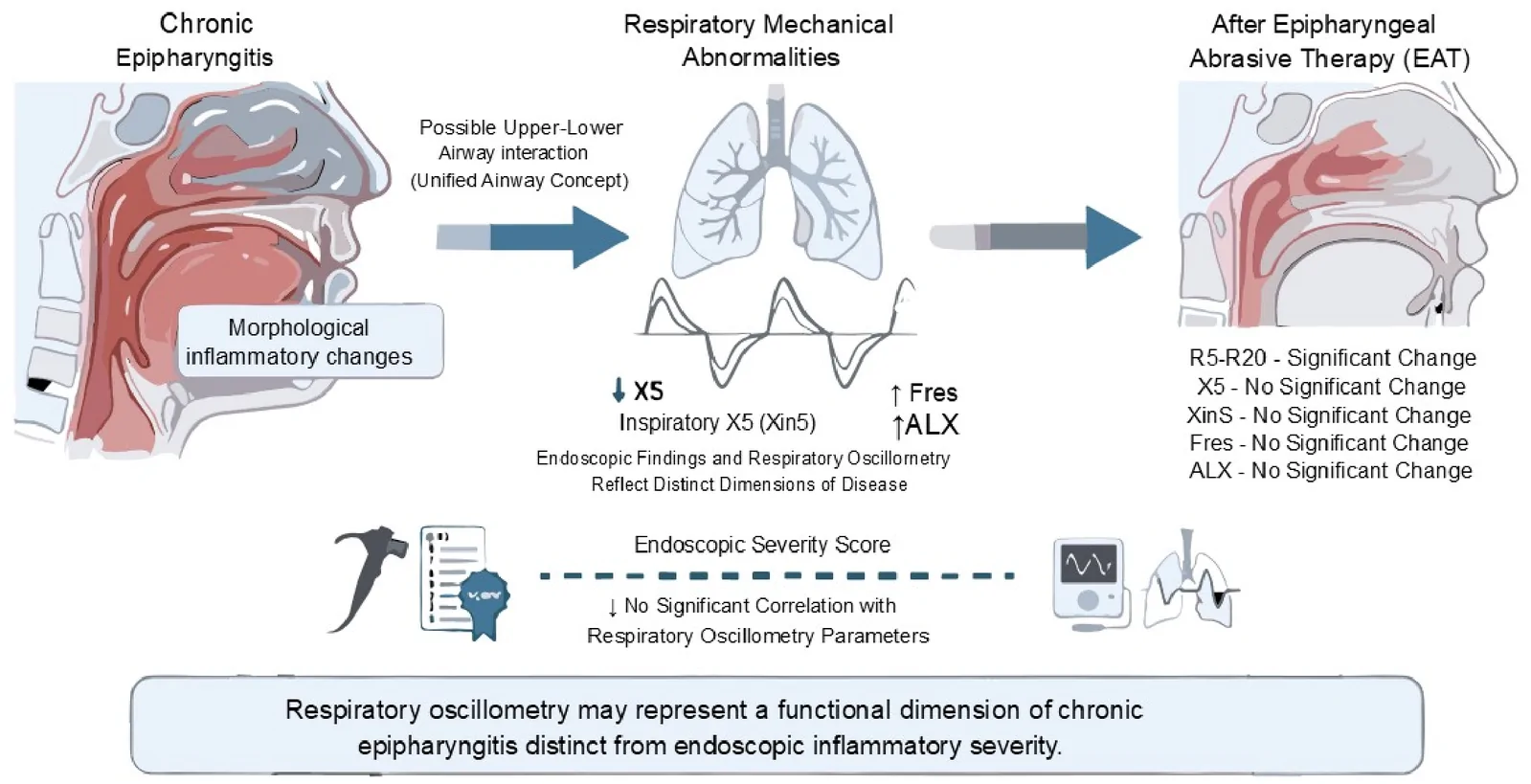
Proposed relationship between chronic epipharyngitis, respiratory mechanical abnormalities, and Epipharyngeal Abrasive Therapy. Conceptual model summarizing the findings of the present study. Patients with chronic epipharyngitis exhibited abnormalities in respiratory oscillometry parameters, including decreased X5 and inspiratory X5 (Xin5) and increased Fres and ALX compared with healthy controls. Following EAT, a significant change was observed only in R5−R20. No significant correlations were identified between endoscopic severity scores and respiratory oscillometry parameters, suggesting that morphological inflammatory findings and functional respiratory abnormalities may represent distinct dimensions of chronic epipharyngitis. Arrows indicate conceptual relationships and directional influences within the proposed model.

Taken together, the present findings suggest that respiratory oscillometry may provide complementary information regarding chronic epipharyngitis that is not fully reflected by endoscopic severity scores. Although the clinical significance of the observed oscillometric abnormalities remains to be clarified, respiratory oscillometry may represent a useful tool for further investigation of the functional aspects of this disease. A conceptual summary of the proposed relationship between chronic epipharyngitis, respiratory mechanical abnormalities, and the effects of Epipharyngeal Abrasive Therapy is shown in Figure 6.

## 5. Conclusions

Patients with chronic epipharyngitis exhibited significant abnormalities in oscillometric reactance parameters compared with healthy controls, suggesting the presence of altered respiratory mechanics despite the absence of clinically recognized lower-airway disease. Although EAT was associated with a significant change in R5−R20, no significant changes were observed in the other oscillometric parameters, and the clinical significance of this finding remains uncertain. Notably, no significant correlations were observed between endoscopic severity scores and respiratory oscillometry parameters. Taken together, these findings suggest that respiratory oscillometry may provide complementary information regarding chronic epipharyngitis; however, the potential clinical implications of EAT require confirmation in larger prospective studies.

## Figures and Tables

**Table 1:** Participant Characteristics.

Variable	Healthy Controls (*n* = 9)	Chronic Epipharyngitis (*n* = 26)
Age, years, median (range)	40 (24–63)	40 (18–72)
Sex	Male 2/female 7	Male 6/female 20

**Table 2:** Distribution of Baseline Endoscopic Severity Scores.

Endoscopic Score	*n* (%)
0	3 (15%)
1	5 (25%)
2	8 (40%)
3	4 (20%)
Total	20 (100%)

**Table 3:** Baseline Respiratory Oscillometry Parameters in Healthy Controls and Patients with Chronic epipharyngitis.

Parameter	Healthy Controls (*n* = 9)	Chronic Epipharyngitis (EAT0, *n* = 26)	*p* Value
R5	2.38 (1.95–2.54)	2.88 (2.28–3.30)	0.1683
R20	2.40 (2.07–2.52)	2.66 (2.19–3.10)	0.1928
R5-R20	0.02 (−0.05–0.13)	0.12 (−0.08–0.36)	0.4732
X5	−0.15 (−0.23–−0.04)	−0.23 (−0.44–−0.16)	0.0344
Xin5	−0.14 (−0.28–−0.06)	−0.33 (−0.55–−0.26)	0.0133
Fres	6.01 (5.31–6.32)	7.10 (6.10–8.39)	0.03
ALX	0.54 (0.27–0.85)	0.78 (0.58–1.55)	0.0362

**Table 4:** Changes in Respiratory Oscillometry Parameters Following Epipharyngeal Abrasive Therapy.

Parameter	EAT0 (*n* = 26)	EAT12 (*n* = 26)	Δ (EAT12−EAT0)	*p* Value
R5	2.88 (2.28–3.30)	2.87 (2.25–3.35)	0.11 (−0.31–0.34)	0.4992
R20	2.66 (2.19–3.10)	2.44 (2.15–2.98)	0.02 (−0.22–0.14)	0.6348
R5-R20	0.12 (−0.08–0.36)	0.29 (−0.03–0.51)	0.04 (−0.03–0.25)	0.0435
X5	−0.23 (−0.44–−0.16)	−0.31 (−0.51–−0.17)	0.02 (−0.18–0.10)	0.671
Xin5	−0.33 (−0.55–−0.26)	−0.38 (−0.68–−0.24)	−0.03 (−0.15–0.15)	0.6348
Fres	7.10 (6.10–8.39)	6.87 (5.91–9.25)	0.18 (−0.66–0.87)	0.4273
ALX	0.78 (0.58–1.55)	0.98 (0.62–1.98)	0.01 (−0.32–0.81)	0.3818

**Table 5:** Correlations Between Baseline Endoscopic Severity Scores and Respiratory Oscillometry Parameters.

Parameter	Spearman r	*p* Value
R5	−0.014	0.946
R20	0.042	0.8389
R5-R20	−0.158	0.4398
X5	0.198	0.3318
Xin5	0.165	0.4192
Fres	−0.263	0.194
ALX	−0.147	0.4745

## Data Availability

The datasets generated and/or analyzed during the current study are available from the author on reasonable request.
